# Whole Volume Apparent Diffusion Coefficient (ADC) Histogram as a Quantitative Imaging Biomarker to Differentiate Breast Lesions: Correlation with the Ki-67 Proliferation Index

**DOI:** 10.1155/2021/4970265

**Published:** 2021-06-24

**Authors:** Yuan Guo, Qing-cong Kong, Li-qi Li, Wen-jie Tang, Wan-li Zhang, Guan-yuan Ning, Jun Xue, Qian-wei Zhou, Ying-ying Liang, Mei Wu, Xin-qing Jiang

**Affiliations:** ^1^The First Affiliated Hospital of Jinan University, Guangzhou, Guangdong, 510630, China; ^2^Department of Radiology, Guangzhou First People's Hospital, South China University of Technology, Guangzhou 510180, China; ^3^Department of Radiology, The Third Affiliated Hospital, Sun Yat-sen University, Guangzhou 510630, China; ^4^Department of Radiology, Guangzhou Red Cross Hospital, Medical College, Jinan University, Guangzhou, Guangdong Province, 510220, China; ^5^University of Hospital, Shanxi University of Finance and Economics, Taiyuan 030600, China; ^6^College of Computer Science and Technology, Zhejiang University of Technology, Hangzhou 310023, China

## Abstract

**Objectives:**

To evaluate the value of the whole volume apparent diffusion coefficient (ADC) histogram in distinguishing between benign and malignant breast lesions and differentiating different molecular subtypes of breast cancers and to assess the correlation between ADC histogram parameters and Ki-67 expression in breast cancers.

**Methods:**

The institutional review board approved this retrospective study. Between September 2016 and February 2019, 189 patients with 84 benign lesions and 105 breast cancers underwent magnetic resonance imaging (MRI). Volumetric ADC histograms were created by placing regions of interest (ROIs) on the whole lesion. The relationships between the ADC parameters and Ki-67 were analysed using Spearman's correlation analysis.

**Results:**

Of the 189 breast lesions included, there were significant differences in patient age (*P* < 0.001) and lesion size (*P* = 0.006) between the benign and malignant lesions. The results also demonstrated significant differences in all ADC histogram parameters between benign and malignant lesions (all *P* < 0.001). The median and mean ADC histogram parameters performed better than the other ADC histogram parameters (AUCs were 0.943 and 0.930, respectively). The receiver operating characteristic (ROC) analysis revealed that the 10th percentile ADC value and entropy could determine the human epidermal growth factor receptor 2 (HER-2) status (both *P* = 0.001) and estrogen receptor (ER)/progesterone receptor (PR) status (*P* = 0.020 and *P* = 0.041, respectively). Among all breast cancer lesions, 35 tumours in the low-proliferation group (Ki − 67 < 14%) and 70 tumours in the high-proliferation group (Ki − 67 ≥ 14) were analysed with ROC curves and correlation analyses. The ROC analysis revealed that entropy and skewness could determine the Ki-67 status (*P* = 0.007 and *P* < 0.001, respectively), and there were weak correlations between ADC entropy (*r* = 0.383) and skewness (*r* = 0.209) and the Ki-67 index.

**Conclusion:**

The volumetric ADC histogram could serve as an imaging marker to determine breast lesion characteristics and may be a supplemental method in predicting tumour proliferation in breast cancer.

## 1. Introduction

Breast cancer is a heterogeneous disease, and the different subtypes can be defined by the immunohistochemical (IHC) approach based on estrogen receptor (ER), progesterone receptor (PR), and human epidermal growth factor receptor 2 (HER-2) and Ki-67 expression levels [1–3]. The accurate preoperative diagnosis of breast lesions and further classification of breast cancer are very important for the selection of an appropriate treatment strategy and prognostic evaluation [4].

The value of diffusion-weighted imaging (DWI) in breast cancer detection and differentiation has already been investigated in a number of previous reports [4–6]. However, the procedure for ADC measurements in breast lesions has not been standardized, and size and positioning of the region of interest (ROI) affect both the ADC levels and reproducibility of the measurements [7]. The ADC measurements in the previous study were mostly based on traditional 2D regions of interest (ROIs) manually drawn from a single representative slice of the breast lesion, which might limit these ADC measurements in their ability to reflect whole tumour characteristics [4, 8–11]. Assessments with whole volume histogram analyses of the ADC might provide more reliable results to reflect the biological characteristics of the heterogeneous breast lesions [3, 8–12].

To the best of our knowledge, the Ki-67 index is considered to represent tumour proliferation status, and a high Ki-67 is associated with an adverse clinical outcome [13]. Ki-67 is helpful for identifying women with early and advanced stages of the disease [14–17], and the change in Ki-67 levels through neoadjuvant therapy has been used as a marker of treatment response recently [13]. Therefore, it makes sense to find a noninvasive imaging biomarker to predict the Ki-67 index.

For all imaging biomarkers, DWI maps and ADC values correlated with tumour cell density, and a low ADC value indicated high cell density or less extracellular space in the histologic analysis. Therefore, the possibility of applying ADC values to predict the Ki-67 index as a prognostic factor has received close attention. In addition, the whole volume ADC histogram could supply more information and predict Ki-67 more accurately than a single ADC value. Some studies analysed the associations between the ADC value and the expression of Ki-67 in breast cancer [7, 18, 19] with different ROI placements; however, data about the relationships between the Ki-67 index and ADC value were inconsistent. Mori et al. reported that there was a moderately significant correlation between the whole tumour ADC histogram (ADC-mean) and Ki-67 [13]. However, Surov et al. found only a weak negative correlation between these two parameters [18]. Some studies found that there were no statistically significant correlations between the ADC value and Ki-67 [7, 19]. Overall, the possibility of using ADC as an imaging marker for proliferation activity in breast cancer is uncertain in clinical practice.

The purpose of the present study was to certify the value of whole volume ADC histograms in differentiating between benign and malignant breast lesions and molecular subtypes of breast cancer and to test the correlation between the ADC histogram parameters and expression of Ki-67 in breast cancer.

## 2. Methods and Materials

### 2.1. Patients

**Figure 1:**
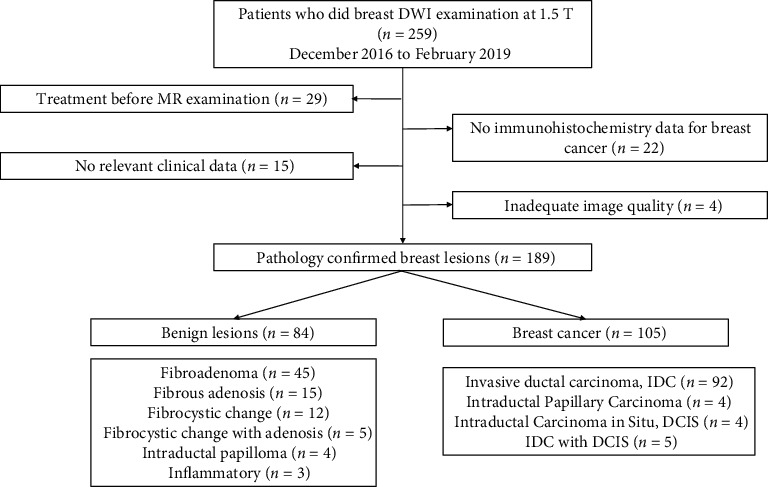
Flowchart of the patient selection process used in this study.

The retrospective, single-centre study was approved by our institutional review board. Between September 2016 and February 2019, 259 patients with suspicious findings on mammography or ultrasound underwent breast MRI at our institution. A total of 189 patients who fulfilled the following inclusion and exclusion criteria were retrospectively evaluated. The inclusion criteria were as follows: (1) patients with pathologically diagnosed breast lesions after surgery or biopsy; (2) all patients who underwent standard breast magnetic resonance imaging, including axial T1-weighted images, fat-suppressed T2-weighted images, axial fat-saturated T1-weighted images pre- and postenhancement, and DWI sequences; and (3) all patients who had complete relevant clinical data; if the patients had breast cancer, immunohistochemistry data and Ki-67 data were needed. The exclusion criteria were as follows: (1) breast-related clinical treatment before MRI and (2) poor image quality due to patient motion, eddy current-induced distortions, or inadequate fat suppression. The patient selection process is demonstrated in Figure 1.

### 2.2. MR Examination Protocol

A total of 189 patients underwent breast MR imaging examinations using a 1.5 T system (uMR 560 1.5 T scanner (United Imaging, Shanghai, China)) with the use of a dedicated four-channel SENSE breast coil. The patients were placed in the prone position with the breasts immobilized. The MRI acquisition protocols were standardized as follows. First, transverse T1-weighted and fat suppressed T2-weighted images were obtained. Second, transverse DWI was performed using a single-shot spin-echo echo-planar imaging sequence with the following parameters: repetition time/echo time (TR/TE), 3800/78 msec; field of view, 350 × 200 mm^2^; matrix, 156 × 156; slice thickness, 4 mm; 27 slices with 0.8 mm gap; voxel size, 2.0 × 2.0 × 4.0 mm^3^; *b* value, 50 and 800 sec/mm^2^; number of averages, 1; and acquisition time, 103 seconds. Third, the gadolinium-based agent Gd-DTPA (gadopentetate dimeglumine, Magnevist; Bayer Healthcare, Berlin, Germany) was intravenously injected at a dose of 0.2 ml/kg of body weight at a rate of 1.5 ml/s, followed by a 20 ml saline flush performed with a high-pressure injector. Axial 3D fat-saturated T1WI were obtained immediately before contrast administration and at six consecutive time points following the administration of the Gd-DTPA contrast agent, with the following parameters: TR/TE, 5.1/2.1 msec; flip angle, 10; field of view, 320 × 320 mm^2^; matrix, 400 × 70; and slice thickness, 2.4 mm. ADC maps were generated with a monoexponential fit for the diffusion data with *b* values of 50 and 800 sec/mm^2^ using the following formula: ADC = [lnS0 − lnS(*b*)]/*b* (where S0 and S(*b*) represent the DWI signal intensity at *b* = 50 and 800 sec/mm^2^, respectively [20, 21]). EPI (fat-suppressed single-shot spin-echo echo-planar imaging) was used for fat suppression.

### 2.3. Imaging Analysis

All DWI scans were retrospectively reviewed by radiologist G.Y. (with 12 years of experience in breast MRI); if the result is questionable or uncertain, the case was discussed with a second senior radiologist to determine by consensus. The radiologist was blinded to the histopathological results. Axial T2-weighted MRI images, dynamic contrast-enhanced images, DWI scans, and ADC maps were transmitted from the workstation to a personal computer for the histogram analysis. The reference for tumour detection was the dynamic contrast-enhanced images and axial T2-weighted images; the largest lesion was chosen for analysis in cases of multifocal or multicentric cancer.

Whole volume ROI placement approaches were applied by each observer (ROI-w): multiple large 2D ROIs were manually drawn on each slice containing the whole lesion of interest and were then combined to create a 3D ROI using the ITK-SNAP tool (http://www.itksnap.org/pmwiki/pmwiki.php). ITK-SNAP is a software application used to segment structures in 3D medical images, and it is free, open-source, and multiplatform. The ROI-w, including any cystic or necrotic portions and haemorrhagic components, was evaluated to assess the heterogeneity of the tumour. The analysis was performed with python software. The ROI containing the whole tumour generated an entire tumour volume reconstruction and displayed the calculated results in the form of a histogram with the Matplotlib package in python. Various ADC histogram parameters were calculated: 10th percentile, mean, 50th percentile (median), 90th percentile, skewness (a measure of asymmetry of the histogram about its mean), kurtosis (a measure of the peakedness of the histogram), and entropy (measure of the variation in the histogram distribution). We followed the methods of Tang et al. [12].

### 2.4. Histopathological Analysis

All patients underwent mastectomy and lumpectomy, and histopathologic evaluations were performed on the resected specimens. All immunohistochemical materials were reassessed in the breast cancer cases, and the findings were confirmed by a dedicated breast pathologist (W.W., with 13 years of experience). The evaluated pathological data included ER, PR, and HER-2 expression and the Ki-67 index. All cases were divided into luminal (luminal A and luminal B) and nonluminal subtypes (HER-2 overexpressed and triple-negative breast cancer).

### 2.5. Statistical Analysis

Statistical analysis was performed using SPSS 21.0 (IBM Corp., Armonk, NY, USA), MedCalc 8 (MedCalc Software, Ostend, Belgium). Levene's test was used to determine whether the continuous variables of the histogram parameters were normally distributed. Continuous variables were compared with Student's *t*-test or Mann–Whitney *U* test if the variables were not normally distributed. Categorical variables were compared using Pearson's chi-squared test or Fisher's exact test. ROC analysis was performed to compare the diagnostic performance of each parameter in distinguishing between benign and malignant breast lesions and different subtypes of breast cancer. Corresponding areas under the ROC curve (AUCs), and the 95% confidence intervals (95% CIs), cut-off value, sensitivity, and specificity are listed. A *P* value ≤ 0.05 was considered statistically significant.

## 3. Results

### 3.1. Patient Characteristics

Of the 189 breast lesions included, 84 (44.4%) were diagnosed as benign and 105 (55.6%) were malignant. The benign and malignant lesion characteristics are shown in Table 1. There was a significant difference in patient age (*P* < 0.001) and lesion size (*P* = 0.006), but there were no significant differences in lesion position (*P* = 0.974), lesion type (*P* = 0.170), and menopausal status (*P* = 0.499) between the benign and malignant breast lesions.

### 3.2. Performance Efficiency of the Whole Volume ADC Histogram in Differentiating between Benign and Malignant Breast Lesions

**Figure 2:**
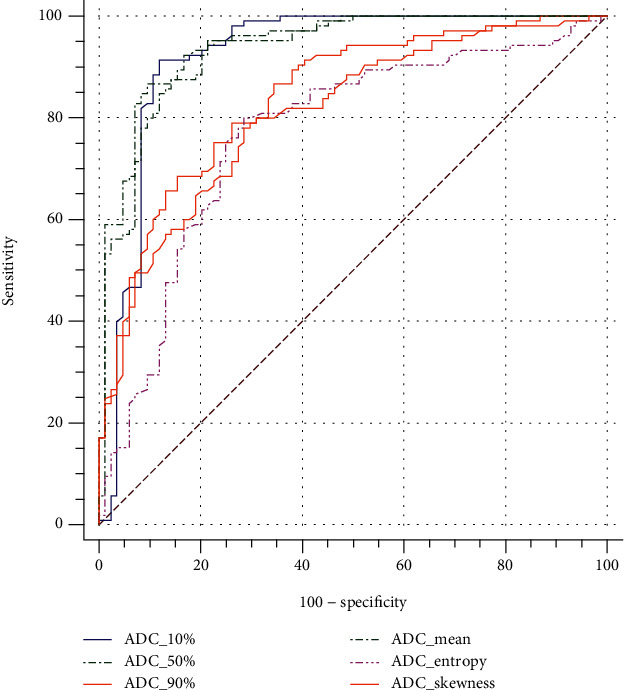
Receiver operating characteristic curve (ROC) for differentiation of benign versus malignant lesions using whole volumetric ADC histogram.

The results demonstrated significant differences in all ADC histogram parameters (including mean, 10th percentile, 50th percentile, 90th percentile, skewness, kurtosis, and entropy) between the benign and malignant lesions (all *P* < 0.001, Table 2). The median and mean ADC histogram parameters performed better than the other ADC parameters (AUC were 0.943 and 0.930, respectively), as shown in Figure 2.

### 3.3. Performance Efficiency of the Whole Volume ADC Histogram in Differentiating between Different Molecular Subtypes of Breast Cancer

The receiver operating characteristic analysis revealed that the 10th percentile whole volume ADC volume and entropy could determine the Her-2 status (*P* = 0.001 and *P* = 0.001, respectively) and ER/PR status (*P* = 0.020 and *P* = 0.041, respectively) (Table 3).

### 3.4. Correlation between the ADC Histogram Parameters and Ki-67 Index

**Figure 3:**
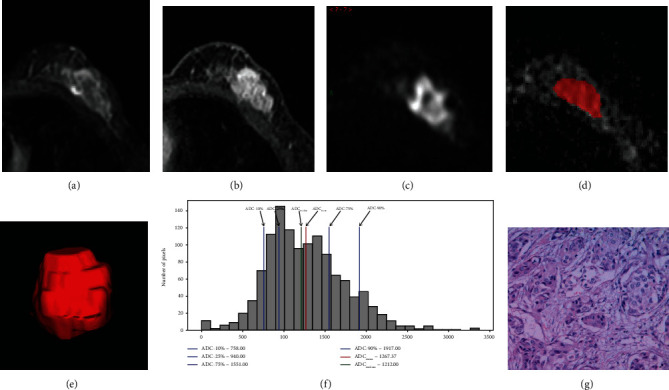
A 64-year-old woman with left breast invasive carcinoma (triple negative breast cancer). T2-weighted imaging (a) shows an irregular left breast mass, 23 mm × 12 mm, with heterogeneous signal. Fat-suppressed contrast-enhanced T1-weighted imaging (b) shows a significant enhancement mass. Diffusion weighted imaging (c) shows high signal mass. (d, e) show the measurement process of whole lesion region of interest (ROI) measurement: manually drawn large 2D-ROIs on each slice (d), then combined multiple 2D-ROI slices to create a 3D-ROI (e). (f) is the whole lesion ADC histogram: ADC_mean_: 1.267; ADC-10%: 0.758; ADC-50%: 1.212; ADC-90%: 1.917 (unit: 10^−3^mm^2^/s); skewness: 1.1; kurtosis: 3.59; entropy: 6.02. (g) HE staining shows left breast invasive carcinoma (HE staining: ×100).

**Figure 4:**
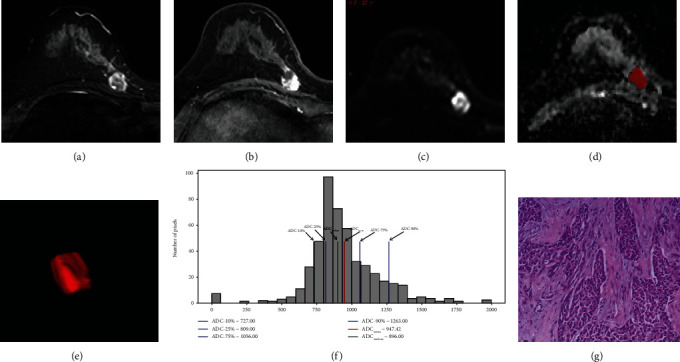
A 62-year-old woman with left breast invasive carcinoma (luminal A). T2-weighted imaging (a) shows a round right breast mass, 18 mm × 12 mm, with heterogeneous signals. Fat-suppressed contrast-enhanced T1-weighted imaging (b) shows a significant heterogeneous enhancement mass. Diffusion weighted imaging (c) shows high signal mass. (d, e) show the measurement process of whole lesion region of interest (ROI) measurement: manually drawn large 2D-ROIs on each slice (d), then combined multiple 2D-ROI slices to create a 3D-ROI (e). (f) is the whole tumour ADC histogram: ADC_mean_: 0.947; ADC-10%: 0.727; ADC-50%: 0.896; ADC-90%: 1.263 (unit: 10^−3^mm^2^/s); skewness: 1.28; kurtosis: 2.61; entropy: 5.89. (g) HE staining shows left breast invasive carcinoma (HE staining: ×100).

For the 105 breast cancer lesions, pathologic evaluation of the Ki-67 ranged from 1 to 86 (median, 45); 35 lesions had a Ki-67 of less than 14 and were categorized as the low-proliferation group (Figure 3), and 70 had a Ki-67 of 14 or greater and were categorized as the high-proliferation group (Figure 4). Receiver operating characteristic analysis revealed that the whole volume ADC entropy and skewness could reflect the Ki-67 status (*P* = 0.007 and *P* < 0.001, respectively) (Table 4).

**Figure 5:**
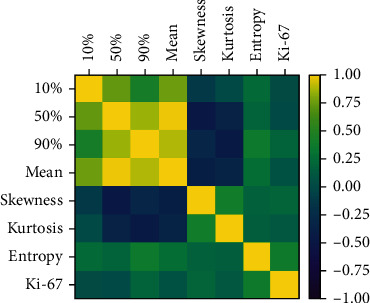
Spearman's rank correlation maps between the parameters of ADC histogram and the Ki-67. The correlation coefficient is indicated with color maps ranging from purple to yellow; purple and yellow indicate the negative and positive strongest correlations, respectively. It shows a weak correlation between ADC entropy and Ki-67.

Spearman's rank correlation maps showed weak correlations between ADC entropy (*r* = 0.383) and skewness (*r* = 0.209) and Ki-67 index (Figure 5), and the 10th, 50th, and 90th percentages had no correlation with Ki-67.

## 4. Discussion

We examined whether the ADC histogram analysis of the whole lesion was reliable and helpful in determining the breast lesion characteristics and whether the ADC histogram parameters were correlated with the Ki-67 index in breast cancer. In this work, the whole volume ADC histogram was used for three purposes: (1) to discriminate between benign and malignant lesions, (2) to assess the molecular subtypes of cancers, and (3) to correlate the ADC parameters with the Ki-67 expression in breast cancer.

The results indicated that the whole lesion ADC histogram exhibited a higher diagnostic performance in distinguishing between benign and malignant breast lesions than between different subtypes of breast cancer, and the ADC histogram showed a relatively higher diagnostic accuracy, such as the 10th percentile ADC value and ADC entropy. In addition, the results showed weak correlations among ADC entropy, skewness, and Ki-67. These results suggest the potential clinical advantage of ADC histograms as imaging markers in diagnosing breast lesions and that ADC histograms may be a supplement for predicting tumour proliferation in breast cancer.

Initially, there were statistically significant differences in the patient age and lesion size between benign and malignant lesions because in our study, most patients were young and had benign lesions, and the masses were relatively small (≤20 mm), which is associated with the sample capacity. Moreover, the value of ADC was emphasized, especially in the histogram-based assessment, which has been used to improve the performance of ADC values in a quantitative manner. Our study showed that the mean, 10th percentile, 50th percentile, and 90th percentile ADC values and skewness, kurtosis, and entropy derived from the whole lesion ADC histogram were able to differentiate between benign and malignant lesions with statistical significance. In malignant lesions, the mean, mode, and percentile ADC values tended to be lower, while the skewness, kurtosis, and entropy values were higher than in benign lesions [10]. The current results were consistent with several previous studies, in which the usefulness of ADC values for providing a differential diagnosis between benign and malignant lesions has been reported, either with 1.5 T or 3.0 T MRI [2]. Therefore, the first aim of our study was to distinguish between benign and malignant breast lesions; then, we paid more attention to differentiating between the molecular subtypes of breast cancer.

In our study, among the whole lesion ADC histograms, the parameters with best discriminative power to differentiate between different molecular subtypes of breast cancer based on ER/PR and HER-2 status were the 10th percentile ADC value and entropy. It is well known that ADC is inversely correlated with tissue cellularity. We assume that a low percentile ADC value based on a whole lesion histogram analysis may accurately define invasive and high cellular density [8]. The region showing the 10th percentile ADC value may reflect the area with the highest cellularity within the tumour, which is highly representative of tumour grade and aggressiveness.

Entropy is a texture-based statistical measure of the variation in the histogram distribution of a given metric and represents the predictability of the intensity of the metric within the tissue. Malignant pathologies tend to affect a tissue heterogeneously and are expected to result in less predictable intensity characteristics within the tissue and thus higher entropy than benign pathologies [9]. In our study, the entropy of Her-2 overexpression and nonluminal breast cancer were higher than those of Her-2-negative and luminal breast cancer, which means that the former had more heterogeneous features.

Ki-67 has been used as a prognostic biomarker for cell proliferation in breast cancer. However, due to the invasive nature of the examination, it could be meaningful in clinical practice to predict the expression of Ki-67 with some noninvasive imaging parameters. The Ki-67 level is evaluated immunohistochemically in the most proliferative area of the tumour and is expected to correlate best with the minimum ADC value and thus is associated with smaller ADC values [13]. In our study, however, none of the 10th to 90th percentile ADC values showed correlations with the Ki-67 index; only skewness and entropy showed a weak correlation with the Ki-67 index. We know that there were different results regarding the correlation between the ADC value and the Ki-67 index. These differences can be explained by several reasons: (1) Mori et al. showed that the 25th, 50th, and 75th percentile and mean values showed similar negative correlations with Ki-67 in invasive breast cancer. We presume that the cause might be the differences in patient cohorts. Only luminal breast cancer was studied by Mori et al., which, compared to nonluminal breast cancer, has less heterogeneous histologic components, with little or no necrotic or degenerative components [13]. In our study, 79 luminal and 26 nonluminal breast cancers were studied, and the heterogeneity of the latter was greater than that of the former. (2) Surov et al. suggested that the ADC value could not be used as a surrogate marker for proliferation activity in breast cancer [18]. However, the threshold of the Ki-67 value was 25% to discriminate between tumours with low Ki-67 expression (<25%) and those with high Ki-67 expression (≥25%). In addition, the traditional manual ROI measurement was assessed. However, this measurement could not reflect the heterogeneity of the whole tumour. In our study, the threshold of the Ki-67 value was 14% for discriminating between tumours, which is the most common threshold. (3) Arponen et al. showed that there was no association between the whole lesion ADC values and the Ki-67 proliferation index [7], and this result was similar to ours. However, only the mean ADC value was calculated in that study, and there were no other parameters. In our study, the 10th to 90th percentile ADC values showed no correlations with the Ki-67 index, but skewness and entropy showed weak correlations with the Ki-67 index. This finding may be explained by the heterogeneous nature of breast cancer, which is correlated with entropy.

We acknowledge several limitations. First, this was a retrospective study. Further prospective and multicentre studies are required to validate our results. Second, our study includes a relatively small number of benign breast lesions and different molecular subtypes of breast cancer. Therefore, it is necessary to expand the database in the future, especially to complement more data on different molecular subtypes of breast cancer, to verify our results. Third, the procedure for ADC measurements in breast lesions should be standardized.

In conclusion, volumetric ADC histograms exhibited a higher diagnostic performance in distinguishing between benign and malignant breast lesions than between different subtypes of breast cancer. The ADC histogram showed a relatively higher diagnostic accuracy than the 10th percentile ADC value and ADC entropy, whereas ADC histogram entropy was weakly correlated with Ki-67. All these results suggest the potential clinical advantage of applying the ADC histogram as an imaging marker in the diagnosis of breast lesions and that the ADC histogram may be a supplemental tool in predicting tumour proliferation in breast cancer.

## Figures and Tables

**Table 1:** Summary of clinical and pathological features of study subjects and tumour characteristics.

	Benign (*n* = 84)	Malignant (*n* = 105)	Statistical value	*P* value
Age (years)	46.97 ± 12.83	55.37 ± 10.61	4.927	<0.001
Position			-0.032	0.974
Left	43 (51.2%)	54 (51.4%)		
Right	41 (48.8%)	51 (48.6%)		
Menstrual status			-0.676	0.499
Premenopausal	28 (33.3%)	40 (38.1%)		
Postmenopausal	56 (66.7%)	65 (61.9%)		
Lesion size			-2.732	0.006
≤20	64 (76.2%)	40 (38.1%)		
>20	20 (23.8%)	65 (61.9%)		
Lesion type			-1.371	0.170
Mass	82 (97.6%)	98 (93.3%)		
Nonmass	2 (2.4%)	7 (6.7%)		

**Table 2:** Comparison of different parameters of whole volumetric ADC histogram ROC curves in differentiation of benign and malignant breast lesions.

Parameter	Benign	Malignant	AUC	95% CI	Sensitivity	Specificity	Cut-off	*P* value
10%	1.259	0.807	0.922	0.874~0.956	91.4	88.1	1.022	<0.001
50%	1.591	1.077	0.943	0.900~0.972	86.7	90.5	1.288	<0.001
90%	1.904	1.457	0.843	0.783~0.891	68.6	84.5	1.615	<0.001
Mean	1.585	1.106	0.930	0.884~0.962	87.6	85.7	1.331	<0.001
Skewness	-0.165	0.601	0.808	0.744~0.861	78.1	71.4	0.160	<0.001
Kurtosis	0.785	1.659	0.705	0.635~0.769	77.1	65.5	0.540	<0.001
Entropy	5.691	6.724	0.768	0.701~0.826	80.0	71.4	5.740	<0.001

**(a):** 

Her-2 status								
Parameter	Her-2(-) (*n* = 75)	Her-2(+) (*n* = 30)	AUC	95% CI	Sensitivity (%)	Specificity (%)	Cut-off	*P* value
ADC-10%	0.779	0.872	0.679	0.581~0.767	80.00	49.33	0.771	0.001
ADC-50%	1.093	1.142	0.585	0.485~0.681	46.67	73.33	1.153	0.165
ADC-90%	1.532	1.571	0.590	0.490~0.685	53.33	70.67	1.587	0.135
Skewness	0.554	0.584	0.505	0.406~0.604	16.67	93.33	1.310	0.934
Kurtosis	1.347	1.791	0.570	0.470~0.667	40.00	78.67	2.170	0.277
Entropy	6.672	7.516	0.680	0.582~0.768	90.00	42.67	6.140	0.001

**(b):** 

ER/PR status								
Parameter	Luminal (*n* = 79)	Nonluminal (*n* = 26)	AUC	95% CI	Sensitivity (%)	Specificity (%)	Cut-off	*P* value
ADC-10%	0.785	0.869	0.642	0.543~0.734	100	24.05	0.665	0.020
ADC-50%	1.090	1.159	0.595	0.495~0.689	42.31	78.48	1.202	0.163
ADC-90%	1.521	1.610	0.574	0.530~0.722	42.30	70.89	1.555	0.258
Skewness	0.539	0.636	0.541	0.441~0.639	19.23	92.41	1.260	0.538
Kurtosis	1.412	1.663	0.532	0.432~0.630	26.92	87.34	3.130	0.643
Entropy	6.738	7.445	0.626	0.526~0.718	92.31	39.24	6.090	0.041

**Table 4:** Comparison of different parameters of whole volumetric ADC histogram ROC curves in differentiation of low or high Ki-67 of breast cancer.

Ki-67 status								
Parameter	<14% (*n* = 35)	≥14% (*n* = 70)	AUC	95% CI	Sensitivity (%)	Specificity (%)	Cut-off	*P* value
ADC-10%	0.809	0.803	0.509	0.409~0.608	88.60	28.60	0.643	0.889
ADC-50%	1.114	1.103	0.532	0.433~0.630	44.29	74.29	1.029	0.591
ADC-90%	1.496	1.471	0.599	0.499~0.693	71.43	45.71	1.422	0.089
Skewness	0.397	0.646	0.647	0.547~0.737	70.00	60.00	0.390	0.007
Kurtosis	1.183	1.620	0.573	0.473~0.669	40.00	80.00	1.730	0.211
Entropy	6.103	7.319	0.746	0.651~0.826	75.71	65.71	6.290	<0.001

## Data Availability

The datasets used and/or analysed during the current study are available from the corresponding author on reasonable request. Anyone who is interested in the information should contact eyguoyuan@scut.edu.cn.
